# Multimorbidity in old age and its impact on life results

**DOI:** 10.1007/s00391-021-01920-9

**Published:** 2021-06-23

**Authors:** Thomas Brijoux, Cristiane Woopen, Susanne Zank

**Affiliations:** 1 cologne center for ethics, rights, economics, and social sciences of health, Universität zu Köln, Cologne, Germany; 2 Lehrstuhl für Rehabilitationswissenschaftliche Gerontologie, Humanwissenschaftliche Fakultät, Universität zu Köln, Cologne, Germany

**Keywords:** Multimorbidity, Oldest old, Depression, Dementia, Quality of life, Multimorbidität, Hochaltrigkeit, Depression, Demenz, Lebensqualität

## Abstract

**Background:**

High prevalence diseases, such as high blood pressure, dementia and depression in old age can lead to multimorbidity, which is often defined as the presence of more than one health condition in an individual. Multimorbidity has negative consequences on health-related quality of life and healthcare utilization. As many age-associated diseases are not curable, therapeutic goals like preservation of autonomy, functioning, and life satisfaction become more important in old age patients.

**Objective:**

The prevalence of multimorbidity dementia and depressive symptoms and the consequences of multimorbidity on autonomy, functioning, and life satisfaction among the oldest old were examined.

**Material and methods:**

In personal computer-assisted interviews, participants of the representative study NRW80+ were asked for which health issues they received medical treatment.

**Results:**

On average, people above the age of 80 years were treated for 3.62 diseases and 31.4% of older people received medical treatment for 5 or more diseases. A connection between multimorbidity and age group could not be shown. Autonomy, functioning, and life satisfaction are reduced in association with multimorbidity.

**Conclusion:**

Multimorbidity is a frequent phenomenon among old people. A lack of diagnostic procedures and medical treatment can be a reason for the missing age trends. The results illustrate the importance of multimorbidity for patient-relevant outcomes and reveal the need to identify patients with multimorbidity.

## Abstract (de)

**Hintergrund:**

Die hohe Prävalenz von Erkrankungen wie Bluthochdruck, Demenz oder depressiven Störungen in der Hochaltrigkeit führt vielfach zu Multimorbidität, dem gleichzeitigen Vorliegen mehrerer Erkrankungen. Multimorbidität wirkt sich negativ auf die gesundheitsbezogene Lebensqualität und Inanspruchnahme von Hilfe aus. Da viele der alterskorrelierten Erkrankungen nicht heilbar sind, werden therapeutische Ziele wie der Erhalt von Funktionalität, Autonomie und Lebensqualität in der Hochaltrigkeit bedeutender.

**Fragestellung:**

Die Häufigkeit von Multimorbidität, demenzieller und depressiver Symptomatik sowie die Auswirkungen von Multimorbidität auf Funktionalität, Autonomie und Lebenszufriedenheit bei über 80-Jährigen wurden untersucht.

**Material und Methode:**

Teilnehmende der repräsentativen Studie NRW80+ wurden in computergestützten persönlichen Interviews befragt, zu welchen gesundheitlichen Problemen sie aktuell ärztlich behandelt werden.

**Ergebnisse:**

Durchschnittlich waren die über 80-Jährigen von 3,62 Erkrankungen betroffen. 31,4 % der Hochaltrigen wurden wegen 5 oder mehr gesundheitlichen Problemen ärztlich behandelt. Zusammenhänge zwischen Alter und Anzahl der behandelten Erkrankungen konnten nicht nachgewiesen werden. Autonomie, Funktionalität und Lebenszufriedenheit sind bei Multimorbidität reduziert.

**Schlussfolgerung:**

Multimorbidität ist in der Hochaltrigkeit ein häufiges Phänomen. Das Fehlen klarer Alterstrends kann auch mit Unterdiagnostik und Unterbehandlung begründet werden. Die Ergebnisse verdeutlichen die Signifikanz von Multimorbidität für patientenrelevante Outcomes. Der Identifikation multimorbider Patienten im Gesundheitssystem kommt eine zentrale Rolle zu.

Aging is often described as a process of gains and losses. While in social and emotional contexts, aging is also associated with gains and plasticity, the emphasis on loss dominates with respect to physical health [30]. Multiple studies like the Berlin aging study (BASE) [ibid.] or the German aging survey [36] have examined the physical health of the older population in Germany and analyzed the most common diagnoses. In persons above the age of 70 years, cardiovascular issues (e.g., hyperlipidemia and cerebral arteriosclerosis) and varicosis are the most common diagnoses according to BASE (ibid.). For people above the age of 75, the Federal Health Monitoring System names high blood pressure and arthrosis as the diseases with the highest prevalence in women and high blood pressure and cardiovascular disease in men [25]. For most diseases, the prevalence is higher in older age groups [25].

Concerning psychiatric disorders, depression and dementia have the highest prevalence in old age [5, 26]. For people aged above 75 years, the prevalence of major depression is 7.2% and 17.1% for subclinical depressive symptoms [19]. In older age groups, prevalence rises for subclinical depressive symptoms (ibid.). Dementia prevalence in old age ranges between 10.7% for men aged 80–84 and 44.8% for women aged above 90 [1]. Prevalence is in general higher for women than for men and higher in older age groups (ibid.). Both diseases, dementia and depression, have a huge impact on people’s lives [25] and influence their quality of life (QoL), their participation in society, their success in geriatric treatments [9] and the management of other diseases [26]. Different aspects of QoL are impacted differently by dementia. While functioning or autonomy are severely reduced [20] the consequences for hedonic measures elements of QoL, especially in self-ratings at the beginning of the disease, are still substantial but of a lesser extent [22].

With higher prevalence for each disease in old age, the number of diseases per person also increases [5, 13]. The coexistence of multiple health conditions is called multimorbidity [14], different approaches exist concerning the number and kind of diseases for the operationalization of multimorbidity [34]. The first large-scale examination of multimorbidity in Germany was done in the Berlin Aging Study [30] and found that 28.0% of the examined persons above the age of 70 were treated for at least 5 diagnoses [30]. The BASE study not only asked the participants for which diagnoses they received medical treatment, participants also underwent detailed diagnostic procedures which allowed statements on undertreatment and underdiagnoses. While 27.6% of men and 25.6% of women aged older than 85 reported 5 or more diseases diagnosed by their general practitioner, 40.9% of men and 54.3% of old age women had 5 or more diseases diagnosed by the study team. These discrepancies were much smaller in persons below the age of 85. In contrast to the cut-off score of five used in BASE, more recent analyses define multimorbidity as the coexistence of two or more diagnoses as reported by participants [14]. In the German aging survey, 57% of the age group of 55–69 reported to be affected by more than 2 self-reported diagnoses [36]. In the German telephone health interview (GEDA) study, 75.4% of women aged between 65 and 74 were affected by 2 health conditions [5]. In women aged 75+, this increased to 81.7%. Fewer multiple health conditions were reported for men (65–74: 68%; 75+: 74.2%) and younger persons [5]. These influences of age and gender have also been shown in a recent international review [32] and are the most frequently studied determinants of multimorbidity [32]. Although multimorbidity serves as a marker for care, dependency, functional impairment, and frailty [7, 37], which occur more frequently among the oldest old, most of these studies analyzed multimorbidity only up to the age of 80. There is, thus, a specific need to look at multimorbidity among older adults.

Multimorbidity among the people aged above 65 years entails higher disability risks as well as poorer health-related QoL and healthcare utilization [21]. In addition, researchers and practitioners have stressed the need to consider multimorbidity explicitly in the healthcare setting [23]. Treating patients with multimorbidity is challenging and specific guidelines have been established to avoid polypharmacy and single disease treatment that cumulatively lead to adverse effects or interactions [2, 31].

The higher portion of incurable chronic diseases also results in a paradigm shift in the therapeutic treatment of geriatric patients. While in younger age, the physical cure of a disease is often the primary therapeutic goal, this moves towards a preservation of functioning, QoL, and autonomy in older age groups [13]. Examinations on the association between QoL and multimorbidity have focused on health-related QoL [18]; however, some evidence shows that multimorbidity is also associated with a decrease in functional health [21] and autonomy [29]. As functioning, QoL, and autonomy are important therapeutic goals in geriatric settings, influences of multimorbidity on these concepts need to be examined for old age.

The theoretical background for this examination is the challenges and potential (CHAPO) model [33] which describes interactions of different facets of QoL, like personal and environmental life chances and life results. Within this model, multimorbidity is understood as a lack of disposable skills and competences, life satisfaction as a result of appreciation of one’s own life, and functioning as well as autonomy as life results in successful life conduct.

The goals of this study were to describe the occurrence of multimorbidity and the most common physical and psychiatric diseases in old age, and to evaluate the influence of multimorbidity on functioning, life satisfaction, and autonomy.

## Methods

Multimorbidity was analyzed with the NRW80+ dataset. The NRW80+ study is a representative study on old people living in North Rhine-Westphalia, Germany’s most populous state, and was conducted from 2016 to 2019. A general description of the NRW80+ study design is given elsewhere [8].

### Variables

Multimorbidity was measured by asking participants if they were medically treated (“Yes” or “No”) for 20 different health issues. The “Yes” answers were counted and interpreted as the number of treated diseases. The list of health issues is derived from the self-administered comorbidity questionnaire [28], the multimorbidity index in age [3], and the list of diseases used by the German aging survey [35].

Mental health conditions assessed in this study include cognition and depression. The DemTect was used to detect cases of dementia and mild cognitive impairment. It is a screening tool for cognitive impairment and shows good classification among the oldest old [15, 17]. We used the cut-off values of 9–12 for mild cognitive impairment and 8 or lower for dementia. The sum scores were derived from age-specific normation of the subtest on people aged 80 and older [17]. In the proxy interviews, the DemTect could not be applied. Proxies were asked to rate the cognitive status of the person they represented on the global deterioration scale [24]. Depressiveness was analyzed by means of the DIA-S4 [12]. The DIA-S4 is a four-item short form of the depression in age scale [11] that is interpreted as the number of depressive symptoms.

Analyzed life results are autonomy, life satisfaction, and functioning. A single item, which could be answered in four points, was used to measure autonomy (“do you arrange your life according to your own ideas?”). Life satisfaction was measured with one item (“all in all, how satisfied are you currently with your life?”) that could be answered in 11 categories between 0 and 10 and had been used similarly in the socioeconomic panel [16]. Functional status was measured with the activities of daily living (ADL) and the instrumental activities of daily living (IADL) subscales of the older Americans resources and services questionnaire [4].

Age and gender were the analyzed sociodemographic variables. Age was analyzed in the 3 age groups 80–84 years, 85–89 years, and above 90 years.

### Sample and bias

We used the full sample of the NRW80+ data, which includes 1687 self-report interviews and 176 proxy interviews. Survey weights are used to balance the sample for age, gender, household size, nursing home status, family status, and region, and to minimize bias in these variables. In the weighted sample 1012 persons are 80–84 years old, 573 respondents are 85–89 years old and 279 are 90 years or older, 676 persons are male 1187 are female.

### Missing values

In this study 2.1% of the data analyzed were missing but missing values exceeded 18.6% in cognition and 7.3% in depression. Complete case reports lead to biased estimators; therefore, missing values were treated via multiple imputation. A total of 20 datasets were imputed, which is sufficient for this missing structure [6]. The presented frequencies and means are the average results of these 20 datasets. Standard deviations for multiple imputed datasets were calculated using Rubin’s formula [27].

### Statistical methods

We present the number of treated diseases in different age and sex groups and use ANOVA and t‑test to explore significant differences between these sociodemographic groups. Linear models with main and interaction terms were used to estimate the relationship between multimorbidity and autonomy, ADL, IADL, and life satisfaction for different age groups and genders. Tests for multicollinearity showed no problems. Heteroscedasticity was addressed by use of robust standard errors HC3 [10]. The analyses were done with SPSS Version 27 (IBM Corp. Released 2020. IBM SPSS Statistics for Windows, Version 27.0. Armonk, NY, USA).

## Results

### Multimorbidity

The mean number of treated diseases per person was 3.62 (Confidence Interval (CI)-95%: 3.51–3.73). Women reported more treated diseases then men (3.73 vs. 3.43, T = 2.74, *p* < 0.01). No treated diagnoses were reported by 4.9% of the sample (women: 5.1%, men 4.6%), 31.4% of the old-aged population reported 5 or more treated diagnoses, while women reported 5 or more treated diseases more frequently than men (33.6% vs. 27.5%) (see Table 1). No uniform influence of age on the number of treated diseases can be shown. In men, the number of treated diseases is highest in the oldest age group (80–84: 3.42; 85–89: 3.36; 90+: 3.66), while in women, the oldest age group reports the least number of treated diseases (80–84: 3.73; 85–89: 3.77; 90+: 3.64). In none of the imputed datasets, did the age group have a significant influence on the number of reported diseases (F = 0.07–0.185, *p* = 0.831–0.993).

**Table 1:** Percentages of the number of treated diseases in different demographic groups

Number of treated diseases	Men (%)			Women (%)		
	80–84 years	85–89 years	90+ years	80–84 years	85–89 years	90+ years
0	4.2	6.1	3	4.9	5.4	4.8
1	17.1	17.3	12	11.8	16.2	10.5
2	17.3	17.5	18	19.4	14.3	22.5
3	19.3	18.6	20.1	17.1	18.2	19
4	15.6	12	16.8	12.8	12.4	10.5
5	10.1	10.5	9	11.6	9	9.9
6	7.3	8.6	9	8.5	9.5	11.1
7	3.3	5.3	4.5	6.5	5.1	6.9
8	2.2	2.5	3	3.1	3.6	2.5
9	1.9	1.6	3	2.3	3.2	0.7
10	0.5	0.1	1.5	1.2	1.9	0.7
11	0.5	0	0	0.8	0.6	0.5
12	0.7	0	0	0	0.8	0.6
13	0	0	0	0	0	0

High blood pressure was the most common treated disease, reported by 59.4% of the sample (60.5% in women and 57.7% in men). Treatment of joint and bone diseases, which were the second most common type of treated diseases, was reported by 45.2%. In joint and bone diseases, a huge gender gap can be observed, with higher prevalence for women (51.5% vs. 35.8%). 34.9% of the subjects reported treatment of heart diseases, which occured more frequently in women than in men (36.5% vs. 32.1%). A full account of the reported treatments is given in Table 2.

**Table 2:** Treated diseases by gender

Reported treatment	Overall (%)		Men (%)		Women (%)	
	Yes	No	Yes	No	Yes	No
Myocardial infection	7.4	92.6	12.5	87.5	4.5	95.5
Heart disease (e.g., heart failure)	34.9	65.2	32.1	67.9	36.5	63.5
Hypertension	59.4	40.5	57.7	42.3	60.4	39.6
Stroke	7.9	92.2	7.1	92.9	8.4	91.6
Mental disorder (e.g., phobia, depression)	6.7	93.3	4.4	95.6	8.1	91.9
Cancer	8	92.0	11.9	88.1	5.8	94.2
Diabetes	16.5	83.6	17.2	82.8	16.1	83.9
Respiratory or lung disease	12.6	87.4	12.8	87.2	12.6	87.4
Back pain	32.6	67.3	27.5	72.5	35.6	64.4
Gastrointestinal disease	13.3	86.8	10.8	89.2	14.7	85.3
Kidney disease	7.3	92.7	8	92	7	93
Liver disease	2	98.3	1.6	98.4	2.2	97.8
Blood disorders (e.g., anemia)	2.5	97.5	2.2	97.8	2.7	97.3
Bone or joint disorder	45.8	54.3	35.8	64.2	51.5	48.5
Urinary disorder	17	83.1	20.2	79.8	15.2	84.8
Insomnia	14.2	85.9	10.8	89.2	16.1	83.9
Vision impairment	30.5	69.7	28.5	71.5	31.5	68.5
Hearing impairment	20.9	79.3	23.7	76.3	19.2	80.8
Neurologic disease	9.9	90.3	8.6	91.4	10.6	89.4
Other chronic disease	12.5	87.3	9.1	90.9	14.5	85.5

### Mental health

Concerning psychiatric diseases, prevalence for problems in cognition as analyzed by DemTect and GDS is 16.5% for dementia and 16% for MCI. About half of the sample (47.5%) shows no depressive symptoms, 24% showed 1 symptom and 28.4% of the people showed 2 or more out of 4 symptoms on the DIA-S4.

### Associations between morbidity and QOL

Multimorbidity influences life satisfaction, autonomy, ADL and IADL. While in each QoL dimension the main effects for age group are significant, most of the interaction terms for multimorbidity and age group are not. All regression coefficients and their corresponding confidence intervals and *p*-values are presented in Table 3.

**Table 3:** Effects of multimorbidity, age group, and gender on functioning, autonomy, and life satisfaction

–	**Autonomy**				**Life satisfaction**			–
*Variable*	*B*	*B-CI-95%*	*T‑value*	*P‑value*	*Beta*	*B CI*	*T‑value*	*P‑value*
Age group: 80–84 (Ref.)								
Age group: 85–89	−0.175	−0.324–−0.025	−2.288	0.022^*^	−0.22	−0.547–−0.107	−1.321	0.107
Age group: 90+	−0.509	−0.770–−0.247	−3.813	<0.001^***^	−1.242	−1.870–−0.613	−3.872	<0.001^***^
Gender: men (Ref.)								
Gender: women	−0.074	−0.221–−0.072	−1.000	0.317	0.126	−0.191–0.443	0.781	0.435
#Treated diseases	−0.044	−0.078–−0.011	−2.577	0.01^*^	−0.165	−0.237–−0.093	−4.502	<0.001^***^
#Treated diseases * Age (85–89)	0.002	−0.036–0.041	0.107	0.915	0.011	−0.073–0.096	0.266	0.79
#Treated diseases * Age (90+)	0.014	−0.053–0.081	0.417	0.677	0.162	0.018–0.306	2.201	0.028*
#Treated diseases * Gender	0.007	−0.046–0.032	0.349	0.727	−0.04	−0.124–0.043	0.781	0.435
Constant	3.771	3.654–3.888	63.026	<0.001^***^	8.55	8.283–8.818	62.655	<0.001^***^
R^2^	–	–	–	0.05	–	–	–	0.05
–	**ADL**				**IADL**			–
*Variable*	*Beta*	*B CI*	*T‑value*	*P‑value*	*Beta*	*B CI*	*T‑value*	*P‑value*
Age group: 80–84 (Ref.)								
Age group: 85–89	−0.207	−0.292–−0.122	−4.785	<0.001^***^	−0.352	−0.461–−0.243	−6.318	<0.001^***^
Age group: 90+	−0.559	−0.720–−0.398	−6.818	<0.001^***^	−0.817	−0.995–−0.638	−8.948	<0.001^***^
Gender: men (Ref.)								
Gender: women	−0.078	−0.159–0.003	−1.892	0.059	−0.13	−0.234–−0.025	−2.432	0.015^*^
#Treated diseases	−0.06	−0.079–−0.040	−5.912	<0.001^***^	−0.075	−0.099–−0.051	−6.088	<0.001^***^
#Treated diseases * Age (85–89)	0.017	−0.004–0.039	1.581	0.114	0.018	−0.008–0.044	1.343	0.179
#Treated diseases * Age (90+)	0.030	−0.008–0.069	1.533	0.125	0.033	−0.007–0.073	1.611	0.107
#Treated diseases * Gender	0.004	−0.018–0.026	0.318	0.751	0.00	−0.027–0.028	−0.018	0.986
Constant	2.002	1.932–2.071	56.150	<0.001^***^	1.942	1.856–2.027	44.465	<0.001^***^
R^2^	–	–	–	0.13	–	–	–	0.20

*#* Number of; *B* regression coefficient; *B CI-95%* 95% confidence intervall of the regression coefficient. Positive values in B indicate higher values in the corresponding outcome when the independent variable increase

^*^p < 0.05, ^**^p < 0.01, ^***^p < 0.001

## Discussion

In this study 31% of old age people report treatment for at least 5 different diagnoses, which is comparable to the rate found in BASE. This study allows a differentiation within the oldest age groups; however, no age-related effects can be observed in this sample. This surprising result can be better understood on the basis of results from the BASE study, where underdiagnoses were most common in the oldest age group. In the NRW80+ study, our operationalization of multimorbidity relies on self-report, which is strongly affected by underdiagnoses [30]. As the NRW80+ study did not involve medical examinations, evidence-based statements about undertreatment and underdiagnoses cannot be made from this sample, indicating the need for studies of the oldest old that include detailed medical diagnostic procedures.

The most frequently reported disease in old age is high blood pressure, which is in line with the results of the Federal Health Monitoring System [25]. Although in depth diagnostic procedures were not feasible in this study the use of the screening tools DemTect and GDS shows that problems with cognition and depressive symptoms are common in old age.

Multimorbidity has a negative impact on the investigated life results, indicating the importance of the construct and the subsequent need to identify patients with multimorbidity in the healthcare setting by use of screening tools. Subsequently, policies that are already a part of guidelines [2], like checks for multimedication and a constant comparison between medical goals and priorities of the patients, need to be put in practice. In all analyzed life results (life satisfaction, autonomy, ADL, and IADL), age effects can be observed. Furthermore, we observe gender differences in IADL which can be explained by higher prevalence rates for dementia among women. The effects of multimorbidity on life satisfaction are reduced in the older age groups which underlines the necessity to differentiate within the oldest age groups in analysis of life satisfaction. Most of the interaction terms remain insignificant, hence it can be assumed that the influence of multimorbidity on autonomy, ADL and IADL does not change in the different age groups.

The usual limitations occur in interpreting cross-sectional data: causal interpretations cannot be made. Multimedication which happens alongside with multimorbidity could not be addressed in this article. In the second wave of the NRW80+ study multimedication is a part of the questionnaire and can be included in future analysis. To examine desired research questions regarding the interactions between different health-related variables, coping behavior, and life results, new study types need to be developed. The questionable circular interactions need to be examined in longitudinal data collected in short time intervals.

## Practical implications

- Multimorbidity affects about one third of the old-aged people in North Rhine-Westphalia.
- Life results like life satisfaction, autonomy, and functioning are associated with multimorbidity. Therefore, diagnostic procedures are needed to identify these patients.
- Further studies are needed to examine undertreatment and underdiagnosis in the oldest old.
